# Yeast goes viral: probing SARS-CoV-2 biology using *S. cerevisiae*

**DOI:** 10.15698/mic2022.04.774

**Published:** 2022-03-21

**Authors:** Brandon Ho, Raphael Loll-Krippleber, Grant W. Brown

**Affiliations:** 1 Department of Biochemistry and Donnelly Centre, University of Toronto, 160 College Street, Toronto, Ontario, Canada M5S3E1.

**Keywords:** SARS-CoV-2, yeast, protein interactions, directed evolution, cell surface display, virus genome assembly

## Abstract

The budding yeast *Saccharomyces cerevisiae* has long been an outstanding platform for understanding the biology of eukaryotic cells. Robust genetics, cell biology, molecular biology, and biochemistry complement deep and detailed genome annotation, a multitude of genome-scale strain collections for functional genomics, and substantial gene conservation with Metazoa to comprise a powerful model for modern biological research. Recently, the yeast model has demonstrated its utility in a perhaps unexpected area, that of eukaryotic virology. Here we discuss three innovative applications of the yeast model system to reveal functions and investigate variants of proteins encoded by the SARS-CoV-2 virus.

The ongoing pandemic has seen an explosion of functional insight into the life cycle of the SARS-CoV-2 coronavirus. Despite the impressive advances that have been made, the precise functions and virus-host interactions of many SARS-CoV-2 proteins remains obscure. The functional significance of variants that arise as the virus evolves is often unknown. We have limited insight into functional alterations that might evolve as the pandemic continues. Finally, establishing means of targeting altered or enhanced viral functions is a priority. Recent papers, including a new study by Klemm *et al.* published in *Microbial Cell* [1], are establishing the utility of the yeast model to address these critical questions.

## UNBIASED FUNCTIONAL ANALYSIS

**Figure 1:**
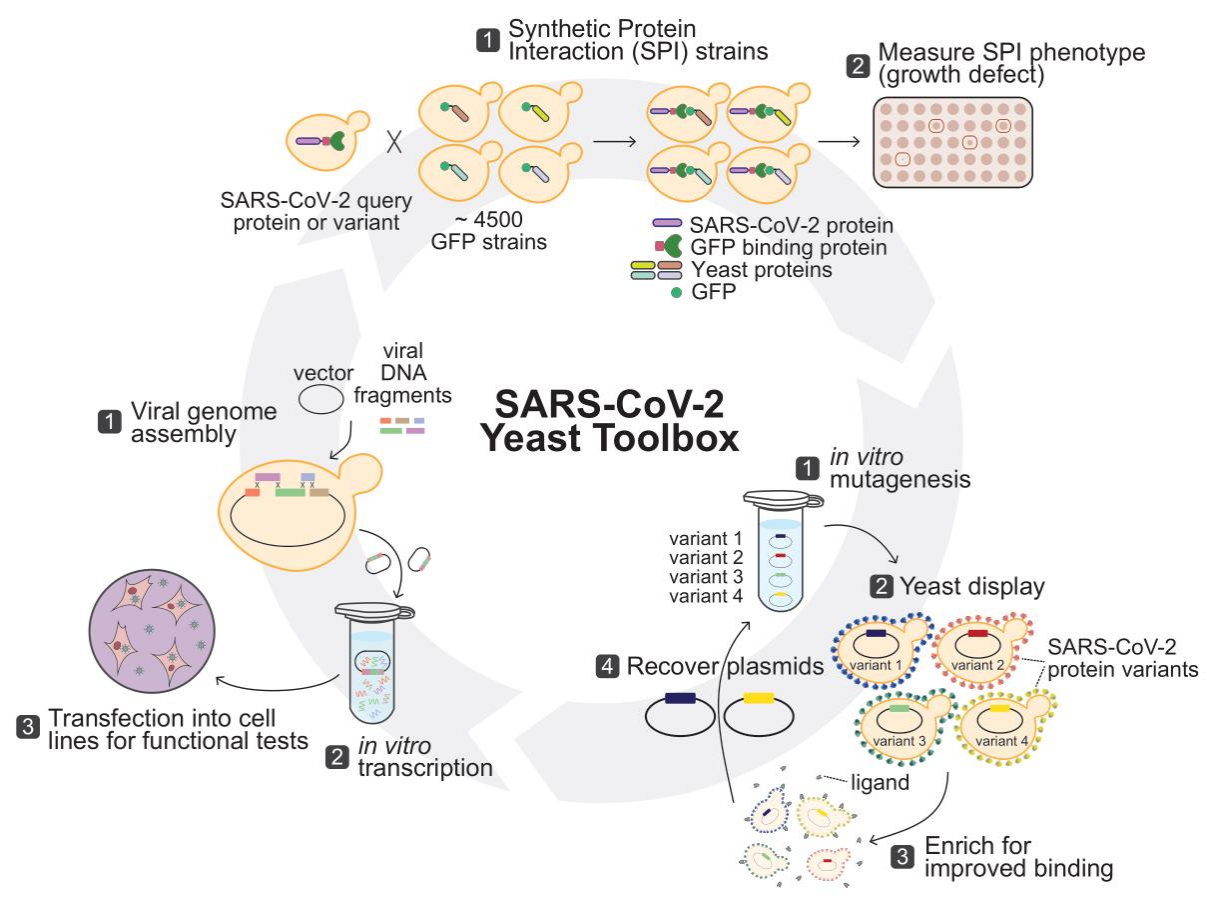
FIGURE 1: Complementary yeast-based technologies to probe SARS-CoV-2 biology. **Top:** Synthetic Protein Interaction (SPI) technology by Klemm et al allows unbiased identification of toxic viral-eukaryotic protein interactions in yeast. Haploid yeast cells expressing a SARS-CoV-2 protein fused to a high affinity GFP binding protein (GPB) are mated to the yeast GFP strain library. Upon expression of the SARS-CoV2-GBP chimera, SPIs are identified as yeast strains having a growth defect due to the forced interaction of the viral query protein with the yeast target protein. **Bottom right:** Directed evolution and yeast display as described by Zahradník et al generates SARS-CoV-2 proteins with enhanced binding properties or activities. A library of protein variants (spike protein, for example) generated by random mutagenesis is displayed on the yeast cell surface. Variants that exhibit improved binding or activity are identified and sorted by flow cytometry, and used to prepare a new variant library. The cycle is repeated until variants with the desired properties are identified. **Bottom left:** Viral genome assembly in yeast as described by Thao et al allows rapid SARS-CoV-2 genome reconstruction and engineering to study viral gene functions. Segments of the SARS-CoV-2 genome are generated via gene synthesis or RT-PCR from patient samples and assembled into a plasmid via recombination in yeast. Upon plasmid recovery, viral RNA is synthesized *in vitro* and electroporated into human cell lines to generate functional viral particles for analysis of virus replication and other viral functions.

Klemm *et al.* present an innovative approach to study the function of SARS-CoV-2 proteins in an unbiased fashion [1] (**Figure 1**). Their approach, termed Synthetic Physical Interaction (SPI), is systematic, and does not depend on prior functional information about the proteins of interest. Importantly, SPI is an *in vivo* functional assay, with a fitness readout that is amenable to quantitative analysis of protein variants and perturbation by small molecules. The basis of SPI is to force an ectopic association between a query protein and each protein in the yeast proteome. Growth defects that result are termed SPIs and indicate that the query protein in specific contexts disrupts the normal homeostasis of the cell [2]. The nature of the cellular pathways that are disrupted by an SPI can provide insight into the function of the query protein [2–5], and provides a simple assay for query protein activity even in the complete absence of existing functional insight for the query protein.

Current uses of SPI have been largely limited to querying yeast protein function but gene and pathway conservation across Eucarya, a feature exploited in many functional assessments using so-called humanized yeast [6], suggesting that SPI could readily be applied for functional analysis of non-yeast proteins. The proteins of the SARS-CoV-2 coronavirus present an exciting test case for this idea. Klemm *et al.* expressed seven SARS-CoV-2 open reading frames spanning a range of functions, both structural and non-structural, and forced their association with endogenous yeast proteins [1]. Growth inhibition by SARS-CoV-2 SPIs was particularly strong, indicating a high potential for viral proteins to disrupt cellular homeostasis. Different SARS-CoV-2 proteins display SPIs with distinct biological processes, reinforcing the idea that SPIs show functional specificity. By focusing on SPIs with yeast proteins having human orthologues, Klemm *et al.* show that SPI can identify human protein complexes known to interact with viral proteins [7] or to be important for viral infection [8, 9], and suggest a novel function for the viral NSP1 in disrupting vesicle trafficking. Thus, SPI presents testable hypotheses about SARS-CoV-2 protein functions and presents a simple cell growth readout of viral protein functions in a model system that is amenable to high-throughput methods to probe structure-function relationships, and to detection of inhibitors of viral protein activities.

## EVOLUTION OF PROTEIN INTERACTIONS

Significant advancements of the yeast cell surface display platform first developed by Schreuder *et al.* in 1993 have made it one of the most widely used methods for directed protein evolution [10]. Combined with *in vitro* random mutagenesis and directed evolution, Zahradnik *et al.* (2021) used yeast display to explore the SARS-CoV-2 mutational landscape and uncover variants of the receptor binding domain (RBD) within the viral spike protein (**Figure 1**), which binds angiotensin-converting enzyme 2 (ACE2) to initiate viral entry [11]. The authors generated libraries of random mutations in the spike RBD and selected variants that resulted in increased spike protein expression at the cell surface or exhibited enhanced binding to ACE2. A comprehensive set of mutations that exhibited high affinity ACE2 binding were identified, mutations that could also confer an adaptive advantage within a human host. Impressively, among the mutations identified with the yeast display system were naturally occurring and clinically relevant S477N, E484K, and N501Y substitutions, demonstrating the capability of this technology to mimic evolution in a shorter timeframe and provide biologically relevant variants. Several novel mutations were also discovered in this study that resulted in the formation of new contacts between the RBD and ACE2 (i.e., Q498R) or new intramolecular interactions that increased RBD stability (i.e., K460 and D420). Therefore, yeast-based directed evolution systems have considerable power to explore the SARS-CoV-2 mutational landscape, with the ability to provide mechanistic insights into current prevalent spike RBD variants while simultaneously predicting viral trajectory and future variants of concern.

## WHOLE VIRUS ASSEMBLY

Viral genome assembly and engineering can prove time consuming and technically challenging using standard molecular cloning methods in *Escherichia coli,* especially for *Coronaviridae* viruses with large genomes [12–14]. Thi Nhu Thao *et al.* (2020) [15] used the highly efficient DNA recombination capabilities of *Saccharomyces cerevisiae* to fully reassemble and genetically engineer the genome of SARS-CoV-2 (**Figure 1**). Using the first full genome sequence of the virus, released in early January 2020, the researchers used Transformation Associated Recombination (TAR) to accurately assemble twelve fragments of synthetic viral DNA, generated via gene synthesis or RT-PCR from patient samples, covering the entire genome of SARS-CoV-2, into a yeast plasmid. The TAR assembly method was concomitantly used to engineer the genome of the virus to express GFP and to introduce small genomic variations corresponding to other human and bat SARS-CoV related viruses. After plasmid recovery from yeast, the assembled viral genomes were transcribed *in vitro* and the resulting RNAs were electroporated into human cell lines. Expression of the viral RNAs was confirmed by the presence of intracellular GFP fluorescence encoded by the assembled viral genomes. Assembly of functional virus particles from the synthetic viral genomes was confirmed by plaque assays. Importantly, the authors were also able to quickly assess the effect of the engineered variants on viral replication. Of particular interest, the introduction of the GFP sequence, via an in-frame insertion in ORF7 which also deleted 242 nucleotides, resulted in slower growth kinetics, demonstrating that this approach facilitates rapid SARS-CoV-2 viral genome engineering and the study of viral gene functions during the complete viral infection cycle.

## THE SARS-CoV-2 YEAST TOOLBOX

These three yeast platforms provide complementary approaches to study SARS-CoV-2 biology. It is not difficult to imagine that the three platforms could be readily combined to produce a comprehensive analysis workflow to probe structure-function relationships in the viral genome (**Figure 1**). SPI and surface display both present ways to quantify the function of individual viral proteins. In the case of SPI, any viral protein could be evaluated and is likely to provoke fitness defects during ectopic interaction with at least a subset of the yeast proteome. The SPIs for a viral protein represent a profile that can be used for analysis of variant forms of the viral protein, where changes in the SPI profile could indicate gains or losses of function, without requiring knowledge of the exact function being queried. Variants for analysis by SPI could originate from circulating SARS-CoV-2 variants, from random or systematic mutagenesis [16], or from directed evolution. Finally, one could envision using the SPI platform with chemical perturbation to identify drugs that inactivate the viral protein and alter its synthetic protein interactions.

Yeast display in combination with directed evolution provides a means of generating variants of viral proteins with enhanced protein-protein interaction properties or activities. Although well suited for viral capsid proteins, it is possible to extend surface display to evolve the strength of any viral protein-protein interaction, be it between two viral proteins or between viral and host protein [17]. Evolved variants could then be rapidly analyzed by SPI to group functionally similar variants and to gain insight into the cellular perturbations that they can cause.

Yeast assembly of SARS-CoV-2 genomes carrying variant virus genes generated and analyzed by evolution and SPI is the gateway to functional analysis of viral gene variants in the context of the complete virus. Clever design of synthetic SARS-CoV-2 to include the modularity of modern synthetic biology approaches [18] could allow variants to be combined for functional analyses at high throughput. Yeast provides a powerful platform for generating and studying SARS-CoV-2 variants from the level of individual proteins to complete virus, one that could be readily applied to other viruses of emerging concern.

## AUTHOR CONTRIBUTION

B.H., R.L.-K., and G.W.B. wrote the commentary.
